# The Chaperone System in Salivary Glands: Hsp90 Prospects for Differential Diagnosis and Treatment of Malignant Tumors

**DOI:** 10.3390/ijms23169317

**Published:** 2022-08-18

**Authors:** Charbel A. Basset, Francesca Rappa, Rosario Barone, Ada Maria Florena, Rossana Porcasi, Everly Conway de Macario, Alberto J. L. Macario, Angelo Leone

**Affiliations:** 1 Department of Biomedicine, Neuroscience and Advanced Diagnostics, Institute of Human Anatomy and Histology, University of Palermo, 90133 Palermo, Italy; 2 Dipartimento di Promozione della Salute, Materno-Infantile, Medicina Interna e Specialistica di Eccellenza “G. D’Alessandro”, University of Palermo, 90133 Palermo, Italy; 3 Euro-Mediterranean Institute of Science and Technology (IEMEST), 90139 Palermo, Italy; 4 Department of Microbiology and Immunology, School of Medicine, University of Maryland at Baltimore-Institute of Marine and Environmental Technology (IMET), Baltimore, MD 21202, USA

**Keywords:** salivary gland tumors, chaperone system, Hsp90, Hsp90 pathogenic, negative chaperonotherapy, Ganetespib, Hsp90 biomarker, differential diagnosis

## Abstract

Salivary gland tumors represent a serious medical problem and new tools for differential diagnosis and patient monitoring are needed. Here, we present data and discuss the potential of molecular chaperones as biomarkers and therapeutic targets, focusing on Hsp10 and Hsp90. The salivary glands are key physiological elements but, unfortunately, the information and the means available for the management of their pathologies, including cancer, are scarce. Progress in the study of carcinogenesis has occurred on various fronts lately, one of which has been the identification of the chaperone system (CS) as a physiological system with presence in all cells and tissues (including the salivary glands) that plays a role in tumor-cell biology. The chief components of the CS are the molecular chaperones, some of which belong to families of evolutionarily related molecules named heat shock protein (Hsp). We are quantifying and mapping these molecular chaperones in salivary glands to determine their possible role in the carcinogenetic mechanisms in these glands and to assess their potential as diagnostic biomarkers and therapeutic targets. Here, we report recent findings on Hsp10 and Hsp90 and show that the quantitative and topographic patterns of tissue Hsp90 are distinctive of malignant tumors and differentiate benign from malignant lesions. The Hsp90 results show a correlation between quantity of chaperone and tumor progression, which in turn calls for negative chaperonotherapy, namely, elimination/inhibition of the chaperone to stop the tumor. We found that in vitro, the Hsp90 inhibitor Ganetespib is cytotoxic for the salivary gland UM-HACC-2A cell line. The drug, by interfering with the pro-survival NF-κB pathway, hampers cellular proliferation and migration, and favors apoptosis, and can, therefore, be considered a suitable candidate for future experimentation to develop a treatment for salivary gland tumors.

## 1. Introduction

Salivary gland tumors are phenotypically heterogeneous and diverse in nature encompassing over 21 different subtypes [1]. While their incidence has been on the rise during the past decades [2,3], they still constitute a rarity among tumors, accounting for 8.1% of all head and neck cancers and 0.5 to 1.2% of all carcinomas [4,5,6]. Consequently, their identification and classification present a major challenge for histopathologists, especially for the untrained in routine microscopy. As a result, misdiagnoses of salivary gland tumors with erroneous identification and/or classification can be expected. Thus, developing novel diagnostic criteria and tools that can be applied without the need of a prerequisite experience in salivary gland histopathology is a necessity.

The chaperone system (CS) is composed of molecular chaperones, chaperone co-factors, co-chaperones, and chaperone interactors and receptors [7]. The CS has canonical functions pertaining to the maintenance of protein homeostasis and, in this, it collaborates with the ubiquitin–proteasome system (UPS) and the chaperone-mediated autophagy machinery. The CS also displays non-canonical functions pertaining to inflammatory and autoimmune conditions and cancer, interacting with the immune system (IS). The molecular chaperones are the chief components of the CS, and they encompass a variety of molecules that can be classified into groups according to molecular weight encompassing the following ranges in kDa: ≤34; 35–54; 55–64; 65–80; 81–99; 100–199; and ≥200 [8]. Some of these groups include families of phylogenetically related molecules named heat shock protein (Hsp), for instance, the Small Hsp (sHsp; which bear the alpha-crystallin motif), Hsp40/DnaJ, Hsp70/DnaK, and Hsp90 families [9]. A proposal for the nomenclature of CS members is available [10]. The CS is a physiological system with presence in all tissues and cells, and many of its components are mobile and migrate in between intercellular compartments and in between cells near and far, interacting with one another [7,11]. Consequently, studying one, or only a few of the CS members in any given tumor, for example, will yield limited information and will not reveal the entire picture of their participation in carcinogenesis. However, it is unfeasible to study all the CS components simultaneously, and therefore, we have been implementing a research plan over the last few years quantifying and mapping various chaperones in salivary glands one step at a time [12,13]. In the work reported here, we investigated Hsp10 and Hsp90.

Molecular chaperones are typically considered cytoprotective, but if abnormal in structure/function/location/concentration, they can become etiopathogenic factors, causing diseases, the chaperonopathies [14]. For example, chaperones have been implicated in cancer development, progression, and metastasization [13,15,16,17,18,19,20,21,22,23,24,25]. The demand for molecular chaperones by cancer cells markedly increases because of their high metabolism and growth rate [26]. There is scarce published information on the role of molecular chaperones in salivary gland tumors. For example, we reported that Hsp27 is differentially expressed in diverse subtypes of salivary gland tumors, possibly playing a role in their pathophysiology [17]. We found a differential pattern of expression of Hsp27 and Hsp60 in Warthin’s tumor (WT) and pleomorphic adenoma (PA) of the submandibular glands (SMG) [12]. However, further investigation is needed to quantify tissue levels and to map the expression of multiple molecular chaperones and other interacting components of the CS in salivary gland tumors to make progress in their management.

Hsp90 has been extensively studied in relation to carcinogenesis because of various reasons, one of which is the nature of its clients, which are kinases involved in proliferative pathways activated in malignancies [27,28]. The NF-κB and PI3K/Akt pathways are among those aberrantly altered in carcinogenesis. Hsp90 is crucial for the stabilization and activation of the IKK complex required for the nuclear translocation of NF-κB [29,30,31]. Hsp90 directly stabilizes and activates both PI3K and Akt, which in turn cross talk with NF-κB allowing its nuclear translocation for gene transcription [32]. We found no information in the scientific literature pertaining to Hsp90/PI3K/Akt/NF-κB pathways in salivary gland tumors. This re-emphasizes the need to investigate their implication in salivary gland carcinogenesis as they may offer a target for therapy complement or alternative to conventional radiotherapy (RT) and chemotherapy (CT). Salivary gland tumors are rare, and so are animal models and suitable cell lines for their study, which slows down progress in their understanding. To date, no protocol for salivary-gland tumor treatment and management has yet been standardized [33,34]. Generally, salivary gland tumors are surgically excised and then surgery may be accompanied by adjuvant RT or CT when deemed necessary [33].

Ganetespib (or STA-9090) is a potent second-generation Hsp90 inhibitor and it was shown to elicit a major anti-tumor response in different cancers [35,36,37,38,39,40,41,42]. Ganetespib was enrolled in 38 clinical studies in which it showed a potent anti-cancer effect and minimal adverse effects (AE) in patients [43,44,45]. Its anti-cancer potential was never tested in salivary gland malignancies.

In this study, we investigated the pattern of expression of two molecular chaperones, Hsp10 and Hsp90, in tumors of the major salivary glands and we examined the effect of Ganetespib on the PI3K/Akt/NF-κB axis in adenoid cystic carcinoma (ACC), aiming to begin identification of efficacious drugs for its treatment.

## 2. Results

### 2.1. Immunohistochemical Assessment of Hsp10 and Hsp90

All results are summarized in Table 1, in which the differences between the benign and malignant pathologies relevant to Hsp90 and presented in detail below, can be visualized.

### 2.2. Hsp10 Levels and Topography in the Tissue of Normal and Pathological Salivary Glands

**Figure 1:**
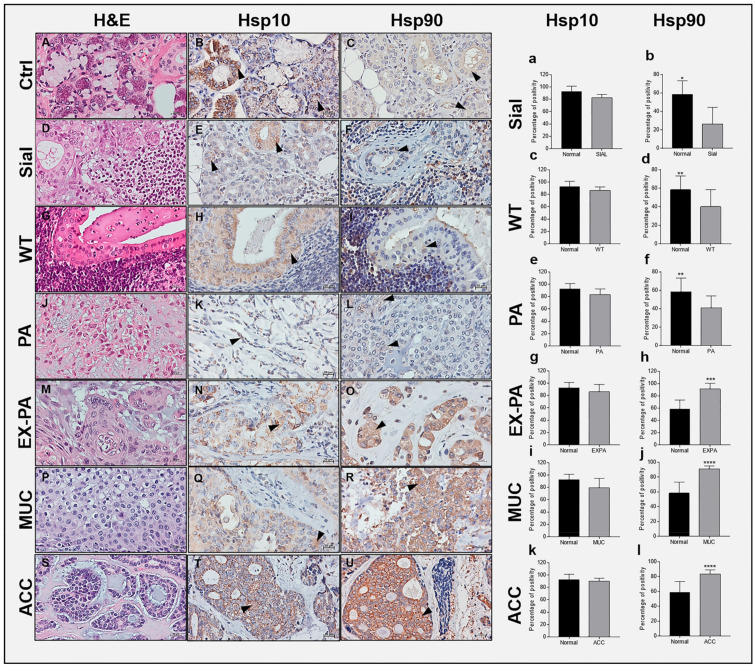
Immunohistochemical determinations of Hsp10 and Hsp90 in adult human SMG and PG. H&E (hematoxylin-eosin staining) of normal (Ctrl) SMG and PG (**A**). Immunostaining of normal SMG and PG shows that Hsp10 (**B**,**a**) and Hsp90 (**C**,**b**) are present in the cytoplasmic portion of the ducts and in the acinar cells (arrowheads). H&E of sialadenitis (Sial) (**D**). Hsp10 (**E**,**a**) and Hsp90 (**F**,**b**) are present in the cytoplasm of the ducts and acini (arrowheads). H&E of Warthin’s tumor (WT) (**G**). Hsp10 (**H**,**c**) and Hsp90 (**I**,**d**) are present in the epithelium of the tumoral tissue (arrowheads). H&E of pleomorphic adenoma (PA) (**J**). Hsp10 (**K**,**e**) and Hsp90 (**L**,**f**) are present in the epithelium of the tumoral tissue (arrowheads). H&E of carcinoma ex-pleomorphic adenoma (EX PA) (**M**). Hsp10 (**N**,**g**) and Hsp90 (**O**,**h**) are strongly positive in the cytoplasm of the neoplastic cells (arrowheads). H&E of mucoepidermoid carcinoma (MUC) (**P**). Hsp10 (**Q**,**i**) and Hsp90 (**R**,**j**) are present in the cytoplasm of the epithelium of the neoplastic cells (arrowheads). H&E of adenoid cystic carcinoma (ACC) (**S**). Hsp10 (**T**,**k**) and Hsp90 (**U**,**l**) occur in the cytoplasm of the neoplastic cells (arrowheads). * Significantly different (*p* ≤ 0.005), ** (*p* ≤ 0.05), *** (*p* ≤ 0.001), **** (*p* ≤ 0.0005). Bar 20 μm.

The immunohistochemical reaction of Hsp10 showed a granular and diffuse cytoplasmic positivity in all specimens (Figure 1). The Hsp10 positivity in the ducts and acini was of high intensity (Figure 1B). A moderate to high intensity was found in the epithelium of the ducts of sialadenitis while the acini displayed a weaker intensity (Figure 1E). In the epithelial component of WT and PA, the immunopositivity was predominantly granular cytoplasmic with a moderate intensity (Figure 1H,K). A moderate to strong granular cytoplasmic Hsp10 positivity was found in the epithelium of the neoplastic cells of EX-PA (Figure 1N), and moderate to intense granular cytoplasmic positivity was found in the cells of MUC (Figure 1Q). In the ACC cancerous cells, the positivity was also granular but more intense than in MUC (Figure 1T). In the histograms to the right, it can be seen that the average percentage of Hsp10 immunopositivity was highest in normal SMG and PG (Figure 1a) with less intensity in sialadenitis (Figure 1a); in the benign tumors WT (Figure 1c) and WT (Figure 1e); and in the malignant tumors EX-PA (Figure 1g), MUC (Figure 1i), and ACC (Figure 1k).

### 2.3. Hsp90 Tissue Levels Decrease in Sialadenitis

**Figure 2:**
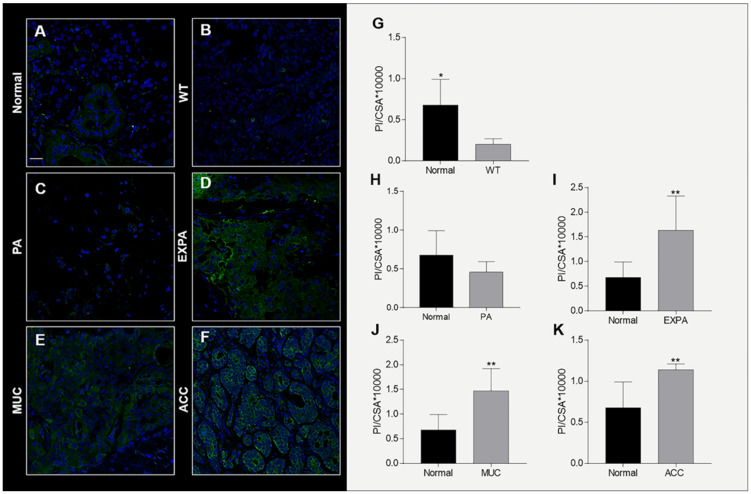
Left. Confocal microscopy analysis for Hsp90 in human adult SMG and PG. Hsp90 immunostaining in Normal (**A**), WT (**B**), PA (**C**), EX-PA (**D**), MUC (**E**), and ACC (**F**). Bar 25 μm. Right. Histogram. The staining intensity for Hsp90 (bars) in Normal (**G**), WT (**G**), PA (**H**), EX-PA (**I**), MUC (**J**), ACC (**K**) was expressed as the mean pixel intensity (PI) normalized to the CSA (cross-sectional area), using the software Leica Application Suite Advanced Fluorescences software. Data are presented as the mean ± SD. * Significantly different (*p* ≤ 0.005), ** (*p* ≤ 0.05). Abbreviations: Normal, normal submandibular and parotid gland; WT, Warthin’s tumor; PA, pleomorphic adenoma; EX-PA, carcinoma ex-pleomorphic adenoma; MUC, mucoepidermoid carcinoma; ACC, adenoid cystic carcinoma.

The Hsp90 reaction showed a diffuse cytoplasmic positivity in all specimens (Figure 1). The Hsp90 positivity observed by IHC and IF in the ducts and acini of normal SMG and PG was of moderate intensity (Figure 1C and Figure 2A). A weak Hsp90 positivity was found in the epithelium of the ducts and acini of sialadenitis (Figure 1F). In this chronic inflammatory condition, there was a decrease in Hsp90 positivity by comparison with normal SMG and PG (*p* = 0.0012) (Figure 1b).

### 2.4. Hsp90 Tissue Levels Decrease in Benign Tumors

In the epithelial component of WT (Figure 1I and Figure 2B) and PA (Figure 1L and Figure 2C), the Hsp90 immunopositivity was predominantly diffuse cytoplasmic with a weak intensity but slightly higher than in sialadenitis. A marked decrease in the Hsp90-immunopositive tumor cells occurred in WT and PA by comparison with normal SMG and PG, as shown by IHC (Figure 1d,f; *p* = 0.0468; *p* = 0.0462) and quantitative IF (Figure 2G,H; *p* = 0.0015; *p* = 0.1594).

### 2.5. Hsp90 Tissue Levels Increase in Malignant Tumors

A strong diffuse cytoplasmic Hsp90 positivity was found in the epithelium of the neoplastic cells of EX-PA (Figure 1O and Figure 2D), MUC (Figure 1R and Figure 2E) and in particular, in the cancerous cells of ACC where the intensity was the strongest (Figure 1U and Figure 2F). A significant increase in Hsp90-immunopositive tumor cells occurred in EX-PA (Figure 1h and Figure 2I; *p* = 0.0006; *p* = 0.0492), MUC ((Figure 1j and Figure 2J; *p* = 0.0004; *p* = 0.0160) and ACC (Figure 1l and Figure 2K; *p* = 0.0002; *p* = 0.0278) relative to the normal SMG and PG tissues, as illustrated by the respective histograms.

### 2.6. Ganetespib Reduces UM-HACC-2A Cell Viability by Inducing Apoptosis

**Figure 3:**
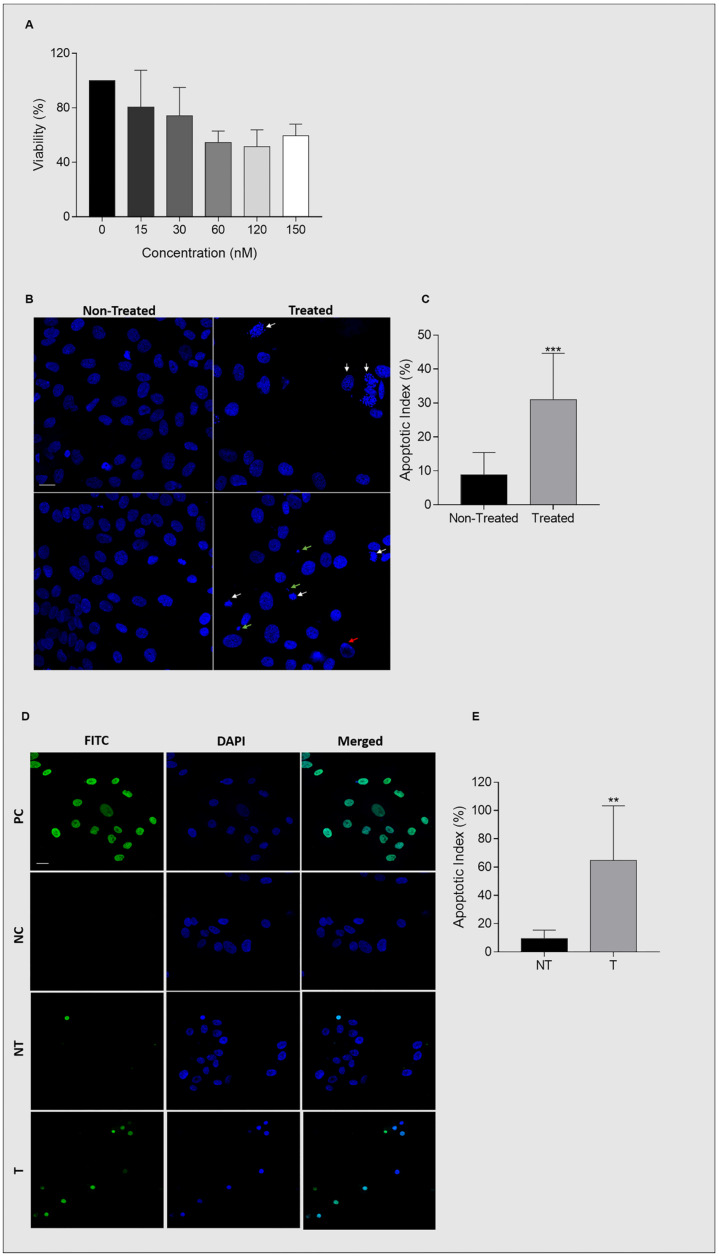
Ganetespib induces apoptosis in UM-HACC-2A cells. (**A**) Histogram representing the percentage of cell viability upon treatment with ascending concentrations of Ganetespib (0, 15, 30, 60, 120, and 150 nM) for 24 h. (**B**) Representative figures of DAPI nuclear staining of Ganetespib-treated and non-treated UM-HACC-2A cells. Apoptosis was assessed by nuclear fragmentation (indicated by white arrows), chromatic condensation (indicated by red arrows), and apoptotic bodies (indicated by green arrows). (**C**) Histogram representing the percentage of apoptotic cells reported as the apoptotic index. (**D**) Representative figures of TUNEL cell death detection assay of Ganetespib-treated and non-treated UM-HACC-2A cells. (**E**) Histogram representing the percentage of apoptotic cells reported as the apoptotic index. Abbreviations: PC, positive control; NC, negative control; NT, non-treated; T, treated. ** Significantly different (*p* ≤ 0.01), *** (*p* ≤ 0.0001). Bar 20 μm.

Ganetespib displayed a cytotoxic effect on UM-HACC-2A cells after 24 h by reducing their viability in a dose-dependent manner from 0 to 60 nM, after which it plateaued at around 50% viability even at 150nM concentration (Figure 3A). Twenty-four hours treatment with 60 nM Ganetespib was selected for the rest of the experiments. Ganetespib induced apoptosis in cells treated with 60 nM concentration for 24 h. Typical morphological features of nuclear apoptosis were assessed by DAPI staining; nuclear fragmentation, chromatin condensation, and apoptotic bodies were apparent in Ganetespib-treated cells (*p* < 0.0001) (Figure 3B,C). These results were confirmed by TUNEL assay that shows apoptosis through DNA strand-breaks labeling. Consistently, Ganetespib-treated cells were more intensely and numerously stained when compared to non-treated cells (*p* = 0.0061) (Figure 3D,E).

### 2.7. Ganetespib Reduces Cell Proliferation and Migration

**Figure 4:**
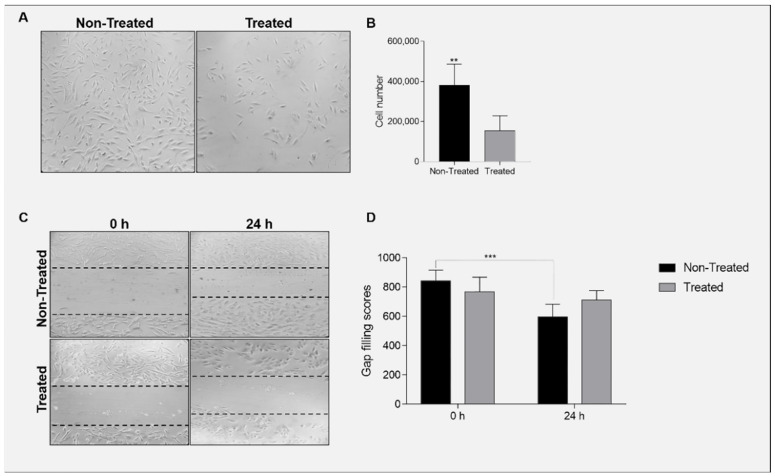
Ganetespib reduces UM-HACC-2A cells proliferation and migration. (**A**) Panel representing non-treated vs. treated cells after 24 h Ganetespib treatment. (**B**) Histogram representing cell counts after 24 h of Ganetespib treatment. (**C**) Panel representing wound-healing assay showing cellular migration at 0 and 24 h after Ganetespib treatment. (**D**) Histogram representing the gap filling scores of cells at 0 and 24 h after Ganetespib treatment. ** Significantly different (*p* ≤ 0.005), *** (*p* ≤ 0.001).

Ganetespib significantly (*p* = 0.0016) reduced cellular proliferation by decreasing UM-HACC-2A cell number (Figure 4A,B). Moreover, Ganetespib significantly reduced cellular migration, as UM-HACC-2A-treated cells were unable to fill the wound in comparison with the non-treated cells (*p* = 0.0005) (Figure 4C,D).

### 2.8. Expression of Hsp90 in Ganetespib-Treated UM-HACC-2A Cells

**Figure 5:**
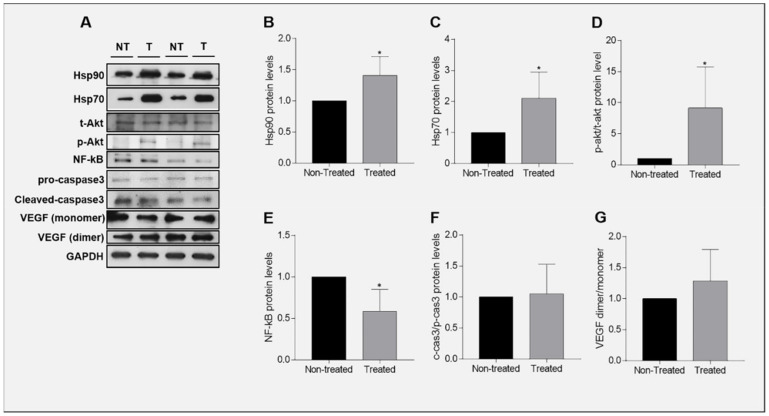
Protein alterations in Ganetespib-treated UM-HACC-2A cells. (**A**) Representative Western blot of protein expression in NT vs. T UM-HACC-2A cells. Histogram representing Hsp90 (**B**), Hsp70 (**C**), p-akt/t-akt (**D**), NF-κB (**E**), c-cas3/p-cas3 (**F**), and VEGF (**G**) protein levels in Ganetespib-treated UM-HACC-2A cells vs. non-treated cells. Abbreviations: NT, non-treated; T, treated; p-ak13hosphorpho Akt; t-akt, total Akt; c-cas3, cleaved caspase 3; p-cas3, pro-caspase 3. * Significantly different (*p* ≤ 0.05).

Ganetespib is a potent second generation Hsp90 inhibitor and its anti-cancer properties has prompted its entry into clinical trials [43,44,45]. However, its effect on the chaperones in adenoid cystic carcinoma of the salivary glands has not yet been reported. After treatment with Ganetespib, Hsp90 (*p* = 0.0350) (Figure 5A,B) and Hsp70 (*p* = 0.0395) (Figure 5A,C) protein levels were found significantly increased in UM-HACC-2A cells compared to the non-treated cells.

### 2.9. Expression of Akt in Ganetespib-Treated UM-HACC-2A Cells

Ganetespib effect on the PI3K/Akt axis has been studied in numerous cancers [45,46,47] but not in salivary gland adenoid cystic carcinomas. We examined the expression of total Akt (t-akt) a hosphorpho-Akt (p-akt) proteins in Ganetespib-treated UM-HACC-2A cells. The results showed that activated Akt (Figure 5A,D) is significantly (*p* = 0.0491) upregulated in Ganetespib-treated UM-HACC-2A cells.

### 2.10. Expression of NF-κB in Ganetespib-Treated UM-HACC-2A Cells

In contrast to the PI3K/Akt axis, scarce information can be found about the effect of Ganetespib on NF-κB in carcinogenesis [48]. Since Hsp90 regulates both Akt and NF-κB, and Akt can activate the NF-κB pathway [32,49,50,51], we sought to examine NF-κB expression in Ganetespib-treated UM-HACC-2A cells. NF-κB was significantly (*p* = 0.0206) downregulated in Ganetespib-treated cells by comparison with the non-treated cells (Figure 5A,E).

### 2.11. Expression of Caspase 3 in Ganetespib-Treated UM-HACC-2A Cells

The mitochondrial cytochrome c-caspase 3 intrinsic apoptotic pathway is an important cell-death pathway [52]. The effect of Ganetespib on caspase 3 activation has been reported in carcinogenesis but not for the salivary gland carcinoma [40,41,53,54,55,56]. We examined the expression of the precursor of caspase 3 (pro-caspase 3) and its activated form (cleaved caspase 3) in Ganetespib-treated UM-HACC-2A cells. The results indicate that both pro-caspase 3 and the activated caspase 3 protein levels are unchanged in Ganetespib-treated cells compared with non-treated cells (*p* = 0.8302) (Figure 5A,F).

### 2.12. Expression of VEGF in Ganetespib-Treated UM-HACC-2A Cells

Vascular endothelial growth factor (VEGF) is a potent cytokine involved in the induction of neovascularization that participates in cellular migration, invasion, and ultimately metastasization [57,58]. A few studies report the effect of Ganetespib on VEGF in cancer but none of them in salivary gland carcinoma [58,59,60]. We examined the expression of VEGF protein monomer and dimer in Ganetespib-treated UM-HACC-2A cells. VEGF levels showed a tendency to increase in Ganaetespib-treated cells by comparison with non-treated cells but this was not statistically significant (*p* = 0.3012) (Figure 5A,G).

## 3. Discussion

Current methods and information are insufficient for proper and rapid diagnosis of tumors of salivary glands and, consequently, patient management is difficult. Our work is a contribution toward remedying this situation for the benefit of health professionals and patients in as much as it adds new criteria for the differential diagnosis of tumors of SMG and PG. We standardized the methods to assess the levels of Hsp10 and Hsp90 in normal and tumoral tissues, and present examples of the type of results the methods provide. This study demonstrates for the first time that assessing Hsp90 can help to discern between malignant and benign tumors of major salivary glands, using two independent immunomorphological techniques, i.e., IF and IHC, with separate analytical methodologies. Our data show that Hsp90 is a promising diagnostic biomarker for salivary gland pathology because it can help differentiate not only between normal and tumorous glands but it can also help discern between normal and inflamed salivary glands, i.e., sialadenitis. Our data show an up-regulation in Hsp90 tissue levels in three types of salivary gland carcinomas, i.e., ACC, MUC, and EX-PA, when compared to normal salivary glands. In contrast, we found a down-regulation in Hsp90 tissue levels in benign salivary gland tumors, WT and PA, and in inflamed salivary glands when compared to their healthy counterpart. Among the four molecular chaperones we have thus far assessed in tumors of salivary glands [12,16] and this work, Hsp90 was the only chaperone that displayed a specific quantitative pattern of expression at the tissue level depending on salivary gland disease, which might indicate that this chaperone plays a role in pathogenesis, including tumorigenesis and inflammation. Based on those results, screening for Hsp90 tissue levels by IF and IHC in patients with inflamed or tumorous salivary glands can be considered useful and should be added to conventional diagnostic tools in pathology units to reduce false positives and increase accuracy of diagnosis.

Hsp90 is considered to be an important molecular chaperone in cancer because of its support of tumor cells and ability to stabilize mutated proto-oncogenes. Consistent with our results, Hsp90 levels did not vary in lobular neoplasia of the breast [61], while Hsp90 positive cells were significantly increased in invasive breast carcinoma [62], again indicating a role of Hsp90 in promoting malignancy. Hsp90 overexpression has been documented as a potential diagnostic marker in hepatocellular carcinoma [63]; cervical [64], colorectal [65], and breast [62] cancers; melanomas [66]; leiomyosarcomas [66]; gastrointestinal stromal tumors (GIST) [66]; and malignant peripheral nerve sheath tumors [66]. Tissue Hsp90 up-regulation was correlated with higher risk GIST and extragastric locations, and served as an independent predictor of recurrence in patients with GIST after complete surgical resection [66]. Hsp90 high IHC scores in oral squamous cell carcinoma (OSCC) [67,68,69], nasopharyngeal carcinoma [70] were associated with lymph node metastasis and a dismal survival rate. Worse OSCC clinical parameters were seen in Hsp90 high-tissue level specimens, which emphasizes the possible role of Hsp90 as a potential independent prognostic biomarker that can effectively predict poor prognosis in OSCC [71]. High Hsp90 expression was associated with worse overall survival in breast cancer, although no correlation between Hsp90 tissue levels and node metastasis was found [72]. Hsp90 increased tissue expression is prominent in melanoma malignancies and metastases when compared with melanocytic nevi [73]. Interestingly, assessing Hsp90 tissue levels was useful in differentiating between low-grade and high-grade ocular surface squamous lesions [74]. Conversely, Hsp90 immunostaining decreased in infiltrative lobular carcinoma confirming once again the specificity of the pattern of expression of Hsps based on tissue, cell, and cancer type [75].

Anti-cancer targeted therapy via Hsp90 inhibition gained significant interest in the last two decades. Geldanamycin (GA) and Radicicol (RC) were the first discovered natural Hsp90 inhibitors that showed promising results in cancer therapy [76,77]. First generation Hsp90 inhibitors GA-derivatives were then synthesized with improved stability, solubility, and binding affinity to Hsp90. These early Hsp90 inhibitors had success in prompting an anti-cancer response and several entered clinical trials. Unfortunately, their severe adverse effects ranging from hepatotoxicity to ocular toxicity resulted in discontinuation of the trials [44]. Ganetespib is a second generation Hsp90 inhibitor with improved affinity and efficiency. In addition, Ganetespib is highly potent at low doses whilst showing minimal adverse effects such as liver toxicity and retinal apoptosis [43,44].

There is a need of salivary gland carcinoma cell lines to make progress in the development of anti-cancer therapies. One available cell line derived from the minor salivary glands adenoid cystic carcinoma is the UM-HACC-2A cell line, established in 2018 [78]. Our study is the first that investigates the effect of an anti-cancer drug such as Ganetespib in this cell line. Ganetespib treatment resulted in an antitumorigenic effect on UM-HACC-2A cells by diminishing cellular proliferation and migration and, furthermore, induced cell death via apoptosis. At 24 h after treatment with Ganetespib, the cells’ viability was approximately 50%, although there was no further reduction at higher doses, i.e., 120 and 150 nM. This could be due to UM-HACC-2A cells possessing the mutant *BRAF* gene that was reported to confer resistance to conventional chemotherapeutic drugs [79,80,81,82].

A body of evidence suggests that carcinoma development is largely linked to accumulation of somatic missense mutations and activation of mutated proto-oncogenes [81]. A prominent example of genetic alterations in cell growth regulatory genes that has been associated with specific colon cancers and melanomas is a single point mutation in the *BRAF* gene [79,80,81,82]. RAF is part of the signaling cascade of the MAPK pathway which regulates cellular growth. RAS binds to RAF (or MAP kinase kinase kinase) and activates it. RAF will in turn phosphorylate MEK (or MAP kinase kinase) that ends up activating ERK (or MAP kinase), allowing its translocation into the nucleus to phosphorylate and activate transcription factors [83]. Mutated BRAF has 10-fold greater kinase activity than wild-type BRAF [80]. Following Hsp90 inhibition, both ERK and Akt activation is dampened but due to mutated BRAF’s increased kinase activity, a rebound activation of Akt and ERK takes place and confers resistance against Hsp90 inhibition by activating mitogenic and survival pathways [82]. This leads us to hypothesize that applying a dual inhibition, or even a triple inhibition, for Akt and/or ERK and Hsp90 simultaneously, may prove to be effective to sensitize carcinomas possessing *BRAF* mutations to Hsp90 inhibitors.

The Ganetespib mechanism of action consists in blocking the Hsp90 ATP binding region on the N-terminus, thus competing with ATP and inhibiting Hsp90, which prompts Hsp90 clients proteins to adopt aberrant conformations, thereby triggering their dephosphorylation and/or UPS-mediated degradation [67,84]. Hsp90 inhibition is characterized by an up-regulation of Hsp70 (a phenomenon called heat shock response), which confirms the success of Hsp90 inhibition. Our data corroborate this since both Hsp90 and Hsp70 proteins were increased as a result of Hsp90 inhibition by Ganetespib in UM-HACC-2A cells. The heat shock response is also reported in OSCC [67], pancreatic cancer [85], hepatocellular carcinoma [86,87], thyroid carcinoma [88] and colon adenocarcinoma [89] cell lines. Hsp90 and Hsp70 form a complex in the cytoplasm and are bound to HSF1, the master stress-inducible transcription factor regulator, keeping its transcriptional activity repressed. Once Hsp90 inhibition occurs, HSF1 is released from the complex and its activity is no longer suppressed, thus enabling its translocation to the nucleus to induce transcription of several Hsp genes, including Hsp90 [67]. This could explain the increase in Hsp90 protein expression post-inhibition. The loss of functional active Hsp90 multichaperone complexes in cancer cells elicit an HSF1-dependent anti-stress response, which confers cell resistance and offsets the cytocidal effects of Hsp90 inhibitors [67,85].

Hsp90 tightly regulates and is indispensable for the stabilization and activation of both Akt and NF-κB pathways [90]. We studied the PI3K-Akt-NF-κB pathway in salivary gland adenoid cystic carcinoma, using the UM-HACC-2A cell line, and our data indicate that activated Akt (p-akt) is upregulated in the Ganetespib-treated cells. Knockdown of Hsp90 was shown to inhibit autophagy by activation of the PI3K/Akt/mTOR pathway in osteosarcoma [91]. The persistence of Akt activation after Hsp90 inhibition may be linked to the UM-HACC-2A cells having the mutant *BRAF* gene. Hsp90 inhibition by AUY922 was shown to inhibit Akt phosphorylation in wild-type *BRAF* colon cancer cells but not in mutant *BRAF* colon cancer cells [82]. While mutant BRAF was shown to be among Hsp90 several clients, Hsp90 inhibition failed to abolish mutant BRAF in colon cancer, suggesting that it may not be solely, nor heavily dependent on Hsp90 for its stabilization [92,93]. CDC37 is a co-chaperone of Hsp90 and has the ability to stabilize mutant BRAF independently of Hsp90 [82]. CDC37 specializes in the co-chaperoning of kinases as it mediates the bridging between Hsp90 and the client kinases before entering the chaperone cycle [94,95]. Stabilization of Akt in mutant *BRAF* colon cancer requires both Hsp90 and CDC37. The combined inhibition of Hsp90 and CDC37 was efficient in abrogating the phosphorylation of Akt in mutant *BRAF* colon cancer cells [82]. Conversely, other studies highlight the suppression of cellular proliferation, migration, and progression through deactivation of the PI3K/Akt pathway by Hsp90 knockdown or inhibition in colorectal [96], gastric [97], osteosarcoma [98], ovarian [99] and cervical [64] cancers. The discrepancy in the results may lie in the type of tissue, type of cancer and the cell lines that were used. Therefore, a strategy involving CDC37 inhibition in combination with Hsp90 inhibitors may be considered to potentiate the effect of Hsp90 inhibitors and combat *BRAF* mutation-acquired resistance.

Our results show that Hsp90 inhibition by Ganetespib in UM-HACC-2A cells downregulated the level of total NF-κB. This was expected, as Hsp90 is a key regulator of the IKK complex. Similar results, in which phosphorylated NF-κB is also downregulated as a consequence of Hsp90 inhibition, were reported for prostate carcinoma [100]. Hsp90 inhibition decreased constitutive and induced NF-κB activity in human myeloid leukemia [49,101,102,103], chronic lymphocytic leukemia [104,105], primary effusion lymphoma [106], and melanoma [107], which led to cytotoxicity; cell-cycle arrest; reduction in proliferation, migration, and invasion; and induced apoptosis. Moreover, blocking NF-κB and Akt survival pathways using the Hsp90 inhibitor potentiates chemotherapeutic drug cytotoxicity in lung cancer [108]. Abolishing activation of the pro-survival NF-κB pathway in UM-HACC-2A cell by Ganetespib may have hindered cellular proliferation and migration and ultimately, led to apoptosis.

## 4. Material and Methods

### 4.1. Ethics Statement

Human salivary gland tissues embedded in paraffin were selected from the archives of the Department of Pathology within the University of Palermo (Italy), Civico Hospital of Palermo (Italy), and Cervello Hospital of Palermo (Italy). All the specimens dated at least 10 years from the moment of the biopsy and therefore, no informed consent from the patients was needed. The study protocol conformed to the ethical guidelines for traceability, collection, transport, conservation, and archiving of cells and tissues for diagnostic investigations of the Pathology Unit issued on May 2015 by the Italian Ministry of Health.

### 4.2. Specimens

A total of 76 cases of formalin-fixed, paraffin-embedded, specimens of parotid (PG) (46) and submandibular (30) glands from healthy controls (n = 10), sialadenitis (n = 13), Wartin’s tumor (n = 12), pleomorphic adenoma (PA) (n = 16), carcinoma ex-pleomorphic adenoma (EX-PA) (n = 8), adenoid cystic carcinoma (ACC) (n = 11), mucoepidermoid carcinoma (MUC) (n = 6), i.e., total n = 76, were obtained from the archives of the Human Pathology section, Department of Health Science, University of Palermo; Unit of pathology, Civico Hospital of Palermo; and Unit of pathology, Cervello Hospital of Palermo, Italy. Human 13 weeks gestation head sections were obtained from the archives of the Department of Anatomy and Histology, University of Palermo, Palermo.

### 4.3. Histopathology

Sections of salivary glands with a thickness of 5 μm were obtained from paraffin blocks and stained with hematoxylin and eosin (H&E) for histological examination. In brief, sections were de-waxed in xylene for 10 min and rehydrated by sequential immersion in decreasing ethanol concentrations. Then, the sections were stained with H&E [109] and analyzed using a Leica DM5000 upright microscope (Leica Microsystems, Heidelberg, Germany). All sections were examined by two independent observers (F.C. and F.R.) in a blind manner, using coded slides and not knowing their source.

### 4.4. Immunohistochemistry

Immunohistochemistry (IHC) reactions for Hsp10 and Hsp90 were carried out on 5–7 μm thick paraffin-embedded tissue sections. The IHC reactions were performed using the automated IHC system (IntelliPath Flx, Biocare Medical, distributed by Bio-Optica, Milan, Italy) of the Biotechnology Laboratory of the Euro-Mediterranean Institute of Sciences and Technologies (IEMEST). The primary antibodies used were anti Hsp10 (mouse monoclonal antibody, Santa Cruz Biotechnology, Dallas, TX, USA, D-8, sc-376313, dilution 1:100) and anti Hsp90 (mouse monoclonal antibody, Santa Cruz Biotechnology, F-8, sc-13119, dilution 1:200). The latter anti-Hsp90 antibody is described by the supplier as able to recognize the two cytosolic Hsp90 isoforms, alpha and beta. Nuclear counterstaining was carried out using hematoxylin (Hematoxylin aqueous formula, DAKO, Golstrup, Denmark. Cat. no. S2020). The slides were then re-dehydrated using ascending concentrations of alcohol followed by complete immersion in xylene. Finally, the slides were prepared for routine microscopy observation with coverslips and an aqueous mounting solution. The observation of the sections was performed with an optical microscope (Leica DM 5000 B, Heidelberg, Germany) connected to a digital camera (Leica DC 300 F). Each tissue section was examined on two separate occasions by two independent observers (FR and FC) to determine the percentage of cells positive for Hsp10 and Hsp90. The evaluation of immunopositivity percentage was calculated in a high-power field (HPF) at 400× of magnification and repeated for 10 HPFs. The average of the percentages of all immuno-quantifications performed in each case for each described group was used for the statistical evaluation. This method of quantification/evaluation was chosen instead of using pertinent software, for example, ImageJ, because software is unable to differentiate between actual positivity and background signal/artifacts, thus reducing the credibility of the results and leading to false positives. Moreover, the software is unable to differentiate between tumor cells and other type of cells (e.g., infiltrating cells). The quantification was conducted by two trained pathologists with extensive experience in chaperonopathies, as described in previous publications from our laboratories [24,110,111,112].

Positive Standards. The positivity of the sections for each of the chaperones was assessed following two main criteria: The percentage of positive cells (≤5% being considered as negative) and the intensity of staining, ranging from + (weak signal intensity) to +++ for the most intense signal, following established procedures [24].

### 4.5. Immunofluorescence and Confocal Microscopy

For immunofluorescence (IF), deparaffinized 5–10 μm salivary gland sections were incubated with antigen-retrieving solution (10 mM tri-sodium citrate, 0.05% Tween-20) for 8 min at 85 °C, and treated with a blocking solution (3% BSA in PBS) for 30 min. Next, the primary antibody anti-Hsp90 mouse monoclonal, Santa Cruz Biotechnology, F-8, sc-13119, dilution 1:50, was applied, and the sections were incubated in a humidified chamber at 4 °C overnight. Then, the sections were incubated for 1 h at 25 °C with a conjugated secondary antibody (anti-mouse IgG-FITC antibody produced in goat, F5897, Sigma-Aldrich, St. Louis, MO, USA). Nuclei were stained with Hoechst Stain Solution (dilution 1:1000, Hoechst 33258, Sigma-Aldrich). The slides were treated with PermaFluor Mountant (Thermo Fisher Scientific, Waltham, MA, USA) and covered with a coverslip. The images were captured using a Leica Confocal Microscope TCS SP8 (Leica Microsystems). Staining intensity for Hsp90 was expressed as the mean pixel intensity (PI) normalized to the CSA (cross-sectional area expressed in pixel) using the software Leica application suite advanced fluorescence software, as previously described [110,111,113].

### 4.6. UM-HACC-2A Cells

Minor salivary gland ACC cells UM-HACC-2A were cultured in Optimal Salivary Gland Medium (OSGM). OSGM consists of high glucose Dulbecco’s Modified Eagle Medium (DMEM) (Corning, Manassas, VA, USA) supplemented with 10% heat-inactivated fetal bovine serum (FBS) (Corning, Woodland, CA, USA), 1% 200 mM (100×) L-Glutamine (Gibco, Life Technologies, Carlsbad, CA, USA), 1% Penicillin/Streptomycin (Gibco, Life Technologies), 0.4 μg/mL Hydrocortisone (StemCell Technologies, Vancouver, BC, Canada), 20 ng/mL recombinant human epidermal growth factor (rhEGF) (Sigma-Aldrich), 5 μg/mL recombinant human insulin (rhInsulin) (Sigma-Aldrich) and 2% Bovine Brain Extract (BBE) (Lonza, Basel, Switzerland). Cells were cultured in un-coated T75 flasks (Corning, Falcon) at 37.0 °C, 5% CO_2_. Cells were passed (1:3) every week using 1 round of 0.25% of trypsin solution (Sigma-Aldrich) for 5 min. Cells were frozen at every passage number in freezing medium containing 8 parts of OSGM, 1 part of dimethylsulfoxide (DMSO) (Sigma-Aldrich) and 1 part of FBS [78].

### 4.7. Cell Viability

Cell viability was assessed using 3-(4,5-dimethylthiazol-2-yl)-2,5-diphenyltetrazolium bromide (MTT) colorimetric assay. One hundred μL of UM-HACC-2A cells were inoculated in a 96 well plate at a density of 2000 cells/well for 24 h to reach confluency. The next day, cells were treated for 24 h with ascending concentrations of Ganetespib/STA-9090 (15 nM, 30 nM, 60 nM, 120 nM and 150 nM) diluted in 5% FBS OSGM deprived of insulin. Following treatment, 100 μL of 0.5 mg/mL MTT (Sigma-Aldrich) was added in each well and incubated for 2 h at 37 °C. The formed formazan crystals were dissolved with 100 μL of DMSO (Sigma-Aldrich) and incubated for 5 min at 37 °C. Absorbance was read at two wavelengths (570 nm and 650 nm), determined with a microplate reader (EZ Read 400, Biochrom, Cambridge, UK).

### 4.8. Wound Healing Assay

UM-HACC-2A cell motility assays were carried out as previously described (Jiali Zhang & Peng, 2009) [114]. Two × 10^5^ cells were seeded in a T25 flask for 24 h and left to reach confluency. The monolayer of cells was scraped using a 1 mL stripette, creating a clear surface area (wound). Then, OSGM was replaced by low serum medium deprived of growth factors (5% FBS and 1% PenStrep) in the control flask and 60 nM of Ganetespib diluted in the same low-serum medium was added in the treated flask and incubated for 24 h. To evaluate the migration of the cells, images of the wound were taken under a light microscope (Leica DMI 3000 B) at 0 time and at intervals of 24 h. The distance of the gap between the two edges of the wound were assessed using ImageJ software.

### 4.9. Cell Proliferation

Two × 10^5^ cells were seeded in a T25 flask for 24 h and left to reach confluency. OSGM was then replaced by 5% FBS OSGM deprived of insulin for the control flask, while OSGM was replaced by 60 nM of Ganetespib diluted in 5% FBS OSGM deprived of insulin in the treated flask. To assess cellular proliferation, the cells were trypsinized and were solubilized in trypan blue and then loaded onto a hemocytometer. The cells were counted under the microscope.

### 4.10. DAPI Staining

One × 10^4^ cells was seeded in a cell culture chamber slide for 48 h and left to reach confluency. OSGM was then replaced by 5% FBS OSGM deprived of insulin in the control well, while OSGM was replaced by 60 nM of Ganetespib diluted in 5% FBS OSGM deprived of insulin in the treated well and incubated for 24 h. After 24-h treatment, cells were washed three times with PBS and then fixed with cold methanol on ice for 30 min followed by two washes with PBS. Then, the cells were stained by DAPI for 15 min in the dark. The stained cells were washed twice with PBS and examined by fluorescence microscopy. Apoptotic cells were identified by chromatin condensation, nuclear fragmentation, and apoptotic bodies. The percentage of apoptotic cells was assessed by calculating the apoptotic index (A.I.) using the following formula: $A.I.=\frac{\mathrm{number}\;\mathrm{of}\;\mathrm{apoptotic}\;\mathrm{cells}}{\mathrm{total}\;\mathrm{number}\;\mathrm{of}\;\mathrm{cells}}\times 100$.

### 4.11. TUNEL Assay

One × 10^4^ cells were seeded in a cell culture chamber slide for 24 h and left to reach confluency. OSGM was then replaced by 5% FBS OSGM deprived of insulin in the non-treated well, while OSGM was replaced by 60 nM of Ganetespib diluted in 5% FBS OSGM deprived of insulin in the treated well and incubated for 24 h. After 24-h treatment, cells were washed one time with PBS and then fixed in 4% paraformaldehyde for 1 h at 25 °C followed by one wash with PBS. Then, the cells were permeabilized for 10 min at 4 °C with permeabilizing solution (0.1% Triton X-100 in 0.1% sodium citrate) and then washed twice with PBS. Positive control was treated with 200 U/mL DNase I for 10 min. Then TUNEL staining was performed using the In Situ Cell Death Detection Kit, Fluorescein (Cat# 11684795910, Roche, Mannheim, Germany). In brief, the cells were incubated with 50 μL of TUNEL reaction mix (terminal deoxynucleotidyl transferase + nucleotide mixture) for 1 h at 37 °C. Negative control cells were incubated with 50 μL of the nucleotide mixture devoid of the terminal deoxynucleotidyl transferase enzyme. Thereafter, cells were washed 3 times with PBS, then mounted with a cover slip and examined by fluorescence microscopy. Apoptotic cells were identified by the stain intensity. The percentage of apoptotic cells was assessed by calculating the apoptotic index (A.I.) using the following formula: $A.I.=\frac{\mathrm{number}\;\mathrm{of}\;\mathrm{intensely}\;\mathrm{stained}\;\mathrm{cells}}{\mathrm{total}\;\mathrm{number}\;\mathrm{of}\;\mathrm{cells}}\times 100$.

### 4.12. Western Blot

Cells were lysed using RIPA buffer containing 0.1% sodium dodecyl sulfate (SDS), 0.5% sodium deoxylate, 10% TRITON X100, 25 mM HEPES, 500 mM DTT, 1.5 mM of magnesium chloride, 300 mM sodium chloride, 200 μM EDTA, 10 mM NaF and 2% of the protease inhibitors. The lysates were centrifuged at 22,000× *g* for 30 min at 4 °C. Protein concentration in the supernatants was measured using the Bradford Protein Assay. For immunoblotting, 60 μg of proteins were separated on 12–15% polyacrylamide gel Electrophoresis (Bio-Rad Laboratory, Hercules, CA, USA) and transferred to nitrocellulose membranes (Bio-Rad Laboratory). The blots were blocked with 5% BSA in Tris-buffered saline and then incubated overnight with rabbit polyclonal anti-Hsp90 (ab13495, 1:1000, Abcam, Cambridge, UK), mouse monoclonal anti-Hsp70 (ab2787, 1:1000, Abcam), rabbit polyclonal anti-cleaved caspase 3 (AB3623, 1:160, Sigma-Aldrich), rabbit polyclonal anti-VEGF (sc152, A-20, 1:500, Santa Cruz, Dallas, TX, USA) rat monoclonal anti-HSF1 (sc-13516, 10H8, 1:500, Santa Cruz), rabbit polyclonal anti-MMP9 (sc-10737, H-129, 1:500, Santa Cruz), rabbit polyclonal anti-NF-κB (sc-372, C-20, 1:200, Santa Cruz), mouse monoclonal anti-Akt1/2/3 (sc-514032, C-11, 1:100, Santa Cruz), rabbit polyclonal anti-GAPDH (ABS16, Sigma-Aldrich). The primary antibodies were detected using horseradish peroxidase–conjugated IgG (rabbit IgG, 1:20,000, GTX26795, Gene Tex, Irvine, CA, USA; mouse IgG, 1:10,000, AP124P, Chemicon International, Temecula, CA, USA; rat IgG, 1:10,000, A9037, Sigma-Aldrich). Bands were visualized by enhanced chemiluminescence. Densitometric analysis was performed using Image J software.

### 4.13. Statistical Evaluation

All data are presented as mean ± standard error. One-way Anova and *t*-test were used for statistical analysis (GraphPad Prism). *p* values equal or less than 0.05 were considered statistically significant.

## Figures and Tables

**Table 1:** Hsp10 and Hsp90 tissue levels in normal and inflamed SMG/PG and their tumors assessed by immunohistochemistry (IHC).

Specimen		Hsp10		Hsp90 ^1^	
		Percentage of Positive Cells	Intensity	Percentage of Positive Cells	Intensity
Normal submandibular (SMG) and parotid (PG) gland	Ducts	96.6	+++	75.5	+/+++
	Acini	92.5	++/+++	36.7	+/++
Sialadenitis	Ducts	90	++/+++	26.4	+/+++
	Acini	75	++	26.4	+/+++
Tumor ^2^	Warthin’s tumor (WT)	86.2	++	40	+/+++
	Pleomorphic adenoma (PA)	83.3	+++	41	+/++
	Carcinoma ex-pleomorphic adenoma (EX-PA)	86	++/+++	91.2	++/+++
	Adenoid cystic carcinoma (ACC)	90	++/+++	83.3	++/+++
	Mucoepidermoid carcinoma (MUC)	79.6	++/+++	91	+++

^1^ The anti-Hsp90 antibody used for IHC is described by the supplier as able to recognize both cytosolic Hsp90 isoforms, alpha and beta. ^2^ Ducts and acini were no discernable in tumor specimens.

## Data Availability

The data presented in this study are available on request from the corresponding author. The data are not publicly available due to our preference in personal interaction with those interested in our work and data and we are open to dialogs with colleagues who identify themselves and show genuine honest interest. We have no problems sharing data in this way.

## Abbreviations

CS	Chaperone system
Hsp	Heat shock protein
UPS	Ubiquitin-proteasome system
IS	Immune system
WT	Warthin’s tumor
PA	Pleomorphic adenoma
SMG	Submandibular gland
RT	Radiotherapy
CT	Chemotherapy
AE	Adverse effects
PG	Parotid gland
EX-PA	Carcinoma ex-pleomorphic adenoma
ACC	Adenoid cystic carcinoma
MUC	Mucoepidermoid carcinoma
H&E	Hematoxylin and Eosin
IHC	Immunohistochemistry
HPF	High-power field
IF	Immunofluorescence
PI	Pixel intensity
cross-sectional area	CSA
OSGM	Optimal salivary gland medium
DMEM	Dulbecco’s modified eagle medium
FBS	Fetal bovine serum
rhEGF	Human epidermal growth factor
BBE	Bovine brain extract
rhInsulin	Recombinant human insulin
DMSO	Dimethylsulfoxide
MTT	3-(4,5-dimethylthiazol-2-yl)-2,5-diphenyltetrazolium bromide
SDS	Sodium dodecyl sulfate
VEGF	Vascular endothelial growth factor
t-akt	Total Akt
p-akt	Phospho-Akt
GIST	Gastrointestinal stromal tumors
OSCC	Oral squamous cell carcinoma
GA	Geldanamycin
RC	Radicicol
